# Data Cleaning in the Evaluation of a Multi-Site Intervention Project

**DOI:** 10.5334/egems.196

**Published:** 2017-12-15

**Authors:** Gavin Welch, Friedrich von Recklinghausen, Andreas Taenzer, Lucy Savitz, Lisa Weiss

**Affiliations:** 1 Maine Medical Center, US; 2 Dartmouth-Hitchcock Medical Center, US; 3 Kaiser Permanente Center for Health Research, US; 4 Dartmouth College, US

**Keywords:** data quality, routinely collected health data, electronic health record, data validity, data error, data completeness

## Abstract

**Context:**

The High Value Healthcare Collaborative (HVHC) sepsis project was a two-year multi-site project where Member health care delivery systems worked on improving sepsis care using a dissemination & implementation framework designed by HVHC. As part of the project evaluation, participating Members provided 5 data submissions over the project period. Members created data files using a uniform specification, but the data sources and methods used to create the data sets differed. Extensive data cleaning was necessary to get a data set usable for the evaluation analysis.

**Case Description:**

HVHC was the coordinating center for the project and received and cleaned all data submissions. Submissions received 3 sequentially more detailed levels of checking by HVHC. The most detailed level evaluated validity by comparing values within-Member over time and between Member. For a subset of episodes Member-submitted data were compared to matched Medicare claims data.

**Findings:**

Inconsistencies in data submissions, particularly for length-of-stay variables were common in early submissions and decreased with subsequent submissions. Multiple resubmissions were sometimes required to get clean data. Data checking also uncovered a systematic difference in the way Medicare and some members defined intensive care unit stay.

**Conclusions:**

Data checking is a critical for ensuring valid analytic results for projects using electronic health record data. It is important to budget sufficient resources for data checking. Interim data submissions and checks help find anomalies early. Data resubmissions should be checked as fixes can introduce new errors. Communicating with those responsible for creating the data set provides critical information.

## Introduction

Evaluating data for errors and omissions is the first step in the analysis phase of any health research project involving data [1]. When a project involves primary data collection, well established methods for preventing and finding errors and ensuring the data collected are consistent with definitions and quality criteria established at the outset of the project are a routine part of project implementation. Multi-center and longitudinal studies with monthly or yearly data collections employ standard data collection protocols and centralized data checking to ensure consistency and quality in data acquisition across centers and over time so that variability and errors due to data collection are minimized. Secondary analysis of research data also benefits from these efforts, although the investigator does not have control over what data was collected or how it was cleaned for its initial purpose. Projects using data abstracted from electronic health records (EHRs) and billing or other administrative systems are using data that was acquired and optimized for clinical or administrative purposes, not research. Opportunities for errors, both systematic and random, in EHR data have been cataloged and included with entreaties that data be cleaned and evaluated for quality before it is analyzed [2,3]. When a project uses secondary EHR and administrative data from multiple sites, with data being pulled independently from different systems at each site, the opportunities for heterogeneity, bias and error are increased, even when each site is using the same specification to extract the data for the project. Because of these extra opportunities for anomalies, checking and cleaning secondary administrative and EHR data is as important, if not more so, than when analyzing primary research data.

The High Value Healthcare Collaborative (HVHC) sepsis project was a two-year multi-site project where Member health care systems worked on improving care of sepsis patients to improve outcomes and decrease cost. The data collection plan for the evaluation of the project included sequential data submissions from each system as the project progressed. Each system used the same data specification to create uniform data sets, but each system also started with unique EHR and administrative databases. The process of checking the submitted data sets for correctness and consistency before they were deemed usable for analysis was a critical part of the analytic process when evaluating the project.

The purpose of this case study is to describe the process that was used to evaluate the submitted data sets prior to analysis and to describe some of the errors we found. Weiskopf and Weng [3] note that how EHR data was checked and cleaned prior to analysis is rarely described in published papers. They argue that this deficit has led to a lack of literature on the quality of EHR data used for research and that greater transparency in this arena could help establish reporting standards for the use of EHR data in research. Our purpose in publishing what we did and what we found is to illustrate the importance of treating EHR and administrative data with the same methods that are routinely applied to primary research data.

## Context

### The HVHC sepsis project

The motivation and purpose for this project are described in more detail elsewhere in this issue [4]. Briefly, the HVHC received grant funding from the Laura and John Arnold Foundation to design a collaborative dissemination framework to broadly disseminate care models within HVHC and beyond. Care for sepsis was chosen as the prototype for developing the framework because of the magnitude of the condition and relative ease in measuring the exposures and outcomes: the care model consisted of a discrete set of evidence-based processes delivered in an in-patient setting and relevant outcomes occurred over relatively short time periods [5]. Primary goals of the project were to increase sepsis treatment bundle compliance (defined below), increase the proportion of sepsis patients discharged to home rather than institution and decrease the cost of sepsis episodes. The project evaluation used a pre-post design with 2015 being the pre-intervention year and 2016 the post-intervention year. The project was reviewed and approved by the Dartmouth-Hitchcock Committee for the Protection of Human Subjects.

### Bundle compliance

A primary outcome measure of this project was compliance with the 3-Hour sepsis bundle, a set of 4 elements to be completed within 3 hours of the “time zero” when a sepsis episode is recognized [6]. The bundle consists of: (1) measuring lactate level; (2) obtaining a blood culture prior to antibiotic administration; (3) administration of broad spectrum antibiotics, and (4) crystalloid fluid resuscitation for patients with clinical signs indicating shock.

### Participation levels

Because of the difficulty and resource intensive nature of the data collection required for this project, HVHC Member hospitals participating in the project could choose to submit data at either of 2 levels of detail. Hospitals providing data at the “simple study” level provided data on bundle element adherence, length of the admission, time in ICU and “time zero.” “Full study” hospitals provided additional data containing dates and times for bundle elements and reasons for non-adherence, lactate and systolic blood pressure values and crystalloid fluid bolus completion time.

## Data collection and preliminary checking

The HVHC Program Management Office (PMO) served as the data coordinating center for the project and performed all data cleaning and analysis on data submitted by Members. The PMO provided participating Member delivery systems with a data specification document containing a case definition, data file formats, skip patterns and a list of legal values for variables. The PMO also held scheduled conference calls with Members and provided a “frequently asked questions” document to clarify the contents of the data specification and answer questions from Members’ staff who were compiling the data files. Five data submissions were scheduled, one for the baseline year and one for each quarter of the intervention year. Each data submission was cumulative in that it included all previously submitted records. Previously submitted records could be corrected on a subsequent submission as needed. A revised data specification was released in March of 2016 that clarified some of the variable definitions and, importantly, changed the length-of-stay (LOS) variable units to hours from days and defining how rounding partial units was to be handled in LOS calculations.

Before data was released to PMO analytic staff for detailed checking and cleaning, preliminary quality checks were carried out by PMO Data Operations and Data Coordination staff. This first round of cleaning included checks that file and variable names were correct; that the appropriate type of data was in each field, for instance date fields contained dates in the prescribed format; that legal values populated categorical variables, and that skip patterns were followed. Counts of missing values were also produced. Member submitted data that were acceptable to the Data Coordinator were evaluated by analytic staff. Details on the process of working with Members to get project data to the PMO is provided by Knowlton et al. [7] in this issue. This manuscript focuses on the data checking that was carried out by PMO analytic staff.

## Case description

The general strategy followed was to prioritize variables that were critical to the analytic plan, either because they were hypothesized to be related to the outcomes of interest or they were potential confounders. We graphically checked univariate distributions both in the whole data set and stratified by Member. We also plotted variables against Medicare paid amount, a primary outcome, on the subset of episodes where that data was available, both in aggregate and at the Member level. Because of the multiple data submissions and the change in LOS unit, we also examined distributions of variables by 3-month calendar quarter to check for unexpected changes in distribution over time. In addition to data cleaning, the quality checks also provided exploratory data analysis to get some insight into the distributions of the variables that could inform later steps in the analysis. For continuous variables, our checks emphasized use of graphical displays and tabular reports over summary measures. This decision made the process more sensitive to outliers and anomalous distributions.

Where possible, we matched episode data from Members to fee-for-service Medicare claims obtained from the Centers for Medicare & Medicaid Services (CMS). We required that patients in matched episodes have fee-for-service Medicare coverage for the entire length of the episode and no zero-cost claims. While only about 8 percent of episodes could be matched, they did provide a subset of records with a source of audited comparison data, albeit for billing, not clinical purposes, for variables that were common to both data sets, including lengths-of-stay and discharge disposition.

The LOS, time between admission and discharge, and intensive care unit LOS (ICU LOS) were included in the data checking process because they were primary cost-drivers for several of the evaluation components. We performed multiple checks on LOS and ICU LOS. First, for each Member, we compared submitted LOS values against rules determined by the data-specification definitions: LOS had to be at least 1 day and could not be less than the reported ICU LOS. ICU LOS values had to be less than or equal to the LOS and could be 0. We also plotted ICU LOS against LOS by Member stratified by calendar quarter.

Plotting the density of the distribution of LOS and ICU LOS allowed us to visually compare the shape of the distributions over time within and between Members. Missing data, as evidenced by sparse or empty plots was also easy to catch using this method. We also compared CMS reported LOS and ICU LOS values to Member submitted values for the subset of records that were matched to CMS claims.

In cases where initial investigations indicated potential anomalies, further investigation was done to verify the existence of the errors and to help the Member’s staff who created the data determine the cause of the problem.

Discharge status was both a predictor of episode cost and a measured outcome for the project. For the CMS-matched episodes, Member-reported and CMS discharge codes were compared. Given the relatively small number of CMS-matched episodes and consistency between CMS and Member data, we did not compare the two measures over time.

The data specification for “full study” participants included variables for systolic blood pressure and lactate levels “closest to sepsis start time.” These variables captured measures of severity of illness. We evaluated the proportion of missing values for both variables by Member.

Other variables, including ZIP Code, which we converted to a measure of socio-economic status, and age were checked for patterns of missing values and anomalous values at the analytic level in addition to the checks they received in Data Operations. In part, the extra checks were motivated by potential changes in anomaly patterns over time due to missing data or inability of a Member to provide data for a certain time period.

## Findings

**Figure 1:**
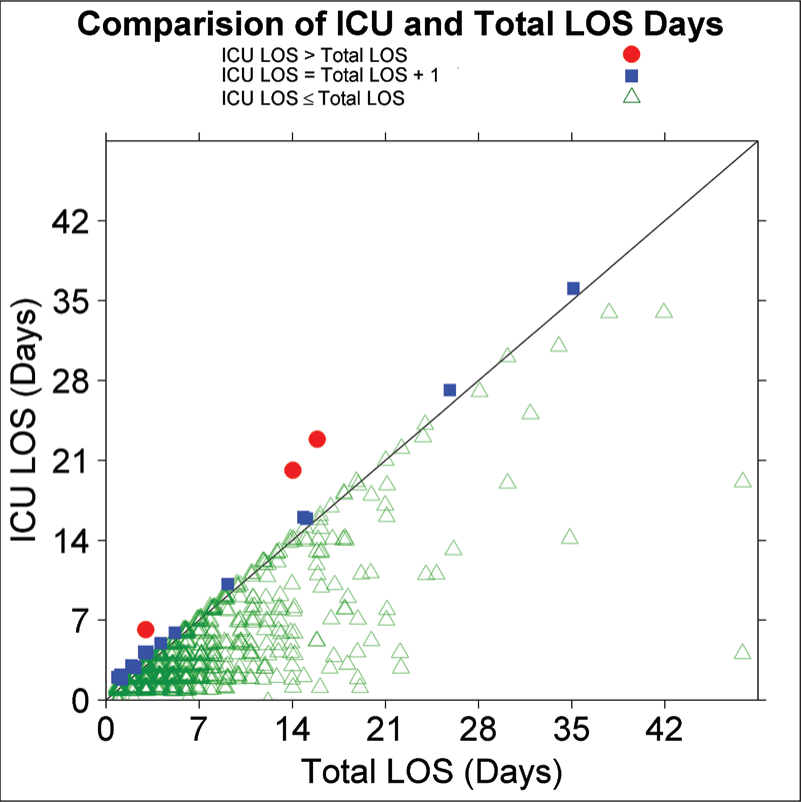
Comparison of the relationship between total length of stay (LOS) and LOS in the intensive care unit (ICU LOS) showing valid and invalid ICU LOS.

Our checks of the length of stay variables found many instances where the reported ICU LOS exceeded the LOS in early data submissions. We also found missing and 0 values for LOS, both in violation of the data specification. An example of the output of a graphical report created to assess the relationship of ICU LOS to LOS is shown in Figure 1. Because the processes used by Members to create the data submissions could change between submissions, we produced reports stratified by calendar (project) quarter. Anomalous ICU LOS values fell into two categories, reporting errors, generally where the ICU LOS was more than one day longer than the LOS, and errors resulting from length of stay calculations using different sources or due to rounding, where the reported ICU LOS was 1 day more than the LOS. Once we identified the 1-day-more error, we explicitly included it in subsequent data checks because of its frequency and likely known causes. While we did not quantify the decrease in these types of errors as the project proceeded, final data sets submitted by Members were free of these types of errors.

**Figure 2:**
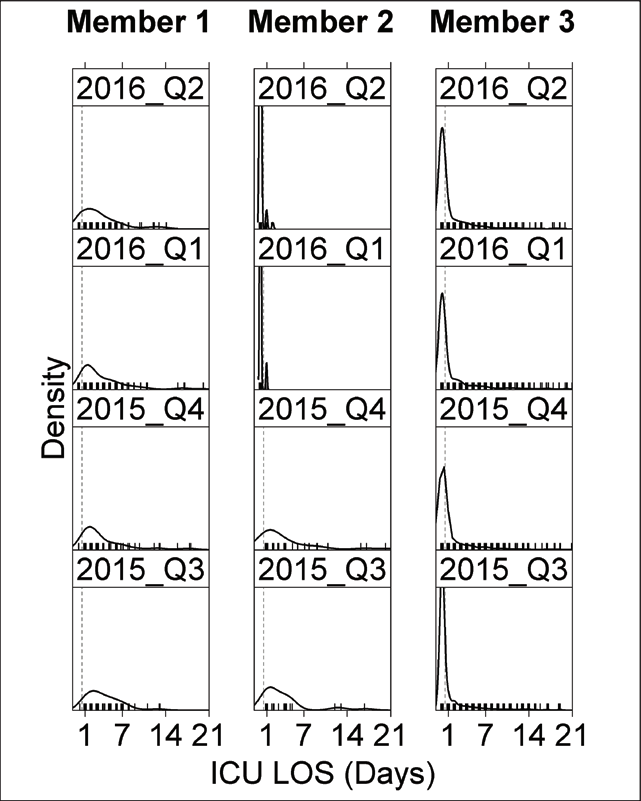
Density plots of intensive care unit length of stay (LOS) for selected Members and project quarters showing anomalies for Member 2.

Our assessment of the density distributions of LOS and ICU LOS was initially carried out for two purposes. First, we wanted to gain some intuition into the shape of the distribution for later statistical analysis. Second, plotting densities served as a quality check for anomalous data. An example plot of ICU LOS by selected sequential project quarters for three participating Members is shown in Figure 2. The difference in shape between 2015 and 2016 for Member 2 was due to a systematic error in the 2016 submissions from that Member that was fixed on a subsequent submission.

Another use of the LOS distributions that became apparent after doing the initial set of data reviews was for quantifying the proportion of cases with 0 days of ICU LOS. This check was a measure of both consistency over time and the types of units (emergency department, general floor or ICU) that were participating within each Member system. Investigating instances of few or no patients without ICU time provided information on how Members were collecting data. In one example that we investigated, a Member was collecting data via manual chart review and had prioritized patients with ICU admissions in the queue of episodes to be abstracted.

Visual inspection of scatter plots comparing Member-provided LOS and ICU LOS with matched CMS values (not shown due to CMS suppression rules) generally indicated a high degree of concordance between Member provided and CMS values for LOS with the exception described below. Because our algorithm for matching CMS claims to sepsis episodes included a 7-day buffer around the Member-provided admission date, we did not expect a perfect match. We also found that plotting data was much more sensitive to anomalies than tabular summaries or summary statistics for these variables.

When comparing Member and CMS values of ICU LOS within Member, we found that CMS ICU LOS values were larger than Member provided values in a subset of episodes for several Members. Our investigation and proposed explanation for that finding is detailed in von Recklinghausen et al. [8] in this issue. We also found one Member who had a large subset of episodes where Member provided ICU LOS was longer than the matched CMS values for a substantial proportion of episodes. The investigation into that issue is still in progress.

**Figure 3:**
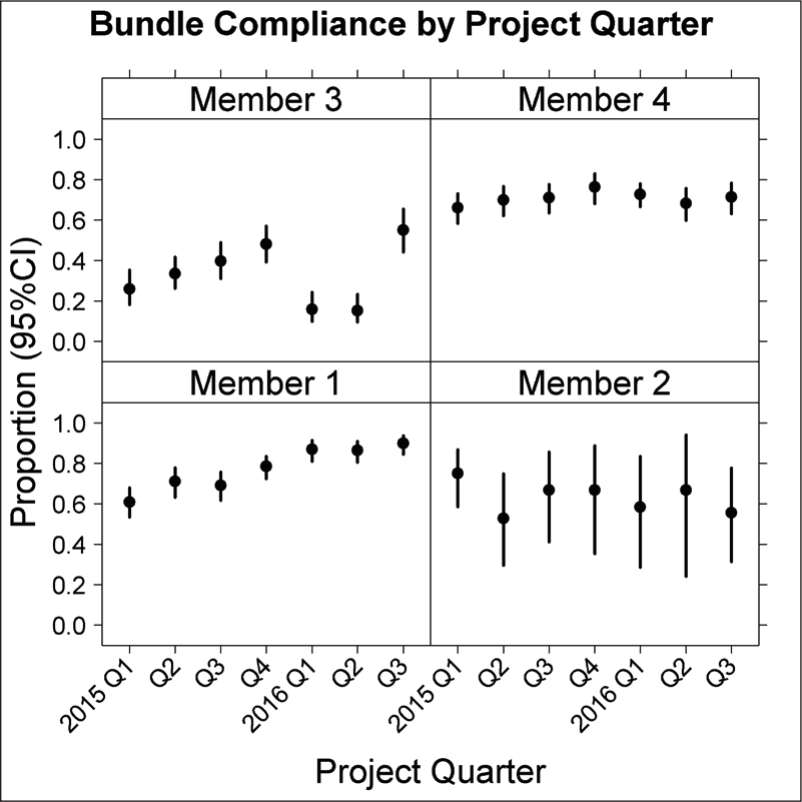
Proportion (95 percent confidence interval) of reported episodes with complete 3-hour sepsis bundle by quarter showing potential anomalies in reporting for Member 3.

We also checked bundle compliance by Member and over time. By plotting the summary data, anomalies were easy to see. Example output showing an unexpected change in percent compliance is shown in Figure 3. Those results were reviewed with the Member and resulted in an updated data submission. Plotting figures similar to Figure 3 showing individual bundle element compliance (not shown) was helpful for diagnosing anomalous bundle compliance findings.

The disease severity measures, presenting lactate and systolic blood pressure, were specified to be collected only by “full study” participants. We found that most “full study” Members provided both variables. However, one “full study” Member could only provide one of the two variables and one “simple study” Member provided both variables. Since these variables were missing by design for a defined portion of Members, we did not investigate anomalies further.

Other variables, including patient age and ZIP Code, were also checked, and, for some Members, large blocks of missing data were found. These anomalies were reviewed with the Member providing the data and, if possible, fixed in a resubmission. In cases where complete data could not be provided, the reasons were documented and accommodation for the missing data was explicitly included in analysis plans.

## Conclusions

Our data checks found anomalies were not uncommon in data sets received by the PMO from Members participating in the project. We found several data submissions with errors sufficiently severe that the Member submitting the data would have been excluded from the analysis had the errors not been fixed. Multiple resubmissions were sometimes required to get a data set that passed all checks and was usable in the evaluation.

The data we collected consisted of a small number of variables and was used to evaluate change in outcomes after an intervention for an acute condition with episodes consisting of one hospitalization. Contrasted with the challenges faced by investigators doing longitudinal comparative effectiveness research on chronic conditions with many more variables and potential threats to validity, for example the case study described by Bayley et al. [9] creating a usable data set for our evaluation would have seemed like a relatively small task. All the same challenges that apply to the more complex project applied here, at scale, however, and substantial time and resources were required to create a credible and valid data-set for the project evaluation.

In investigating potential anomalies, we learned from data staff at Member systems that a wide variety of methods were used to create the submitted data sets. Some Members abstracted records by hand, others pulled from existing condition-specific registries or abstracted from combinations of EHR and administrative databases. There were also examples where resource constraints limited the type and number of episodes provided or where there were constraints on the accuracy or availability of a data element. All of this information is useful for correctly interpreting the results of the project and it illustrates the value of gathering information on the methods used by sites in multi-site studies to create the data sets they submit. Additionally, our investigation into discrepancies between Member reported ICU LOS and Medicare ICU LOS would have been impossible without cooperation from Member staff.

Our data cleaning was able to find anomalies that were detectable using comparisons within and between Members. We relied heavily on consistency in our checks. We were not able to catch consistent under-reporting, for instance if a Member did not implement the case definition correctly or systematically did not provide data on all eligible episodes.

Similarly, methods used and decisions made by Members’ staff when data sets were created as part of the extract, transform, load process involved potential sources of error [10], any errors of this type not generating discernible discrepancies on data checking would go undetected using our methods. Examples of procedures to mitigate this type of error at the data-submitter level have been published for other HVHC projects [11].

In addition to the many frameworks, recommendations and examples that have been published regarding the use of clinical data for research, we contribute the following 4 pragmatic, and perhaps self evident, recommendations based on our experience:

1. Plan and budget sufficient time and personnel for data checking.
2. Run interim data through prototype analyses as early in the project as possible. It’s difficult to anticipate all possible data errors *a priori*, but errors that are found via preliminary analysis before the end of the data collection period can be fixed.
3. Do not assume that a resubmitted data set has been fixed. Fixes can introduce new errors, so run the whole suite of evaluations against both new and resubmitted data sets.
4. Communicating directly with the staff creating the data at Member systems is invaluable. They can provide critical insight into the provenance of the data, how complete it is and the methods used to collect it. All of this information can help ensure that the appropriate data is used in an appropriate analysis.

### Validity of observational data and publishing data cleaning methods

The validity of conclusions in observational research is established by considering, eliminating or reducing sources of bias that could detract from or affect results [12]. Ensuring that the data used in research or evaluation is as complete and correct as possible is an important step in eliminating wrong, incomplete or biased data as an alternative explanation for observed results.

The problem of data quality in EHR and other non-research data is not obscure. There is an extensive literature on the importance of evaluating and reporting data quality [3,13]. Examples specifically targeted at comparative effectiveness research [14] as well as more abstract discussions [10] are also quite prevalent. From a research policy perspective, Benchimol et al. [15] proposed reporting recommendations called REporting of studies Conducted using Observational Routinely-collected Data (RECORD) that extend the STrengthening the Reporting of OBservational studies in Epidemiology (STROBE) guidelines [16]. RECORD Item 12.2 states: “Authors should provide information on the data cleaning methods used in the study.” Kahn et al. [17] note that what they term data quality activities are often study-specific, “one off” and not well documented. They propose a vocabulary and framework to help alleviate this problem.

More generally, and like other aspects of observational research, one set of data checks and one set of findings is essentially a sample from the universe of investigations and results, no two are exactly the same. However, as more investigators contribute data quality publications to the literature, the more opportunities there will be to discern helpful patterns that can be used in future projects using EHR data. We came to the problem of multi-site EHR data quality from the perspective of a coordinating center with the ability to compare data across participating systems. By presenting the data checks that we did and some of our results, as well as the process we used to investigate the anomalies we found, we hope to contribute to the discussion of the need for more open sharing of data cleaning practices and findings.
